# Association between genetic polymorphisms and osteonecrosis in steroid treatment populations: a detailed stratified and dose-response meta-analysis

**DOI:** 10.1042/BSR20190024

**Published:** 2019-05-14

**Authors:** Jun Yang, Ming Jing, Xiaoge Yang

**Affiliations:** Department of Orthopedics & Traumatology, Yuxi Municipal Hospital of TCM, Yunnan Province 653100, P.R. China

**Keywords:** meta analysis, single nucleotide polymorphisms, steroids

## Abstract

Steroid treatment has become recognized as an important risk factor for avascular osteonecrosis of the femoral head. However, not all patients who receive long-term, high-dose steroids develop osteonecrosis, indicating that there are individual differences in occurrence.

We explored the relationship between polymorphisms and steroid-induced osteonecrosis of the femoral head (SONFH) incidence with variables. We used a multilevel mixed-effects logistic regression model, which is an expansion of logistic regression, for each type of steroid, primary disease, drug dose, applied duration, and single-nucleotide polymorphism (SNP). We also conducted a dose-response meta-analysis to analyze the cumulative dosage and SONFH risk in mutation carriers. There were significant correlations between the ABCB1 rs1045642 mutant and SONFH in the prednisone-use and methylprednisolone/prednisone-use populations. The ABCB1 rs2032582 mutant homozygote had a protective effect in the methylprednisolone/prednisolone renal transplant population. For ApoB rs693, mutation increased the incidence of SONFH in prednisone-use and methylprednisolone/prednisolone-use populations and renal transplant patients. For ApoB rs1042031, mutation increased the risk of SONFH in the prednisone-use population. The PAI-1 rs1799768 mutation had a protective effect on the SONFH risk prednisone-use and renal transplant populations. ABCB1 rs1045642 mutations have a protective effect against SONFH, and ApoB rs693 and rs1042031 increase the SONFH risk. Cumulative dosage and treatment duration had little effect on the results. In addition, there was a dose-effect correlation in ABCB1 rs1045642 and rs2032582 mutation carriers.

## Introduction

Steroid-induced osteonecrosis of the femoral head (SONFH), which leads to collapse of the femoral head and articular dysfunction, has an incidence of 9–40% amongst patients receiving steroid treatment [1]. The exact pathology of SONFH is still unclear and might be related to lipid metabolism disorders, abnormal microcirculation, insufficient blood supply, inflammation, and bone marrow mesenchymal stem cell osteogenesis differentiation dysfunction. The abnormal blood supply leads to the apoptosis of osteocytes and osteoblasts, followed by bone loss and reduced bone mineral density [2]. Lipid metabolism dysfunction is also an important pathology, and steroid application may lead to an increase in blood lipid levels and then to the development of intravascular lipid embolism in the microvasculature [3]. Embolism accumulation might affect microcirculation and increase the pressure of the intramedullary cavity, eventually leading to the death of bone cells. Long-term or mass steroid application is the critical pathogenesis of SONFH [4]. However, not all patients who receive long-term, high-dose steroids will develop SONFH, indicating that there are individual differences in the occurrence of SONFH.

To date, several meta-analyses have been published and shown that PAI-1 4G/5G (rs1799768) [5], ABCB1 C3435T (rs1045642) [5–7] and CYP3A activity [8] are associated with SONFH incidence. However, the results of the ABCB1 G2677T/A polymorphism are still disputed [5–7]. Our previous research reported only the single-nucleotide polymorphisms (SNPs) that appeared in more than three studies and indicated that ABCB1 rs1045642 has a protective effect on SONFH in an allelic model and that the ApoB rs693 and rs1042031 mutations promote the pathogenesis of SONFH. ABCB1 rs2032582, MTHFR rs1801133, and PAI-1 rs1799768 were not correlated with SONFH incidence. However, heterogeneity still exists in the previous results, and further analysis of the characteristics of the included studies is needed. Therefore, this research further analyzes the effects of primary disease, type of steroids, cumulative steroid dosage, and treatment duration on the results.

## Methods

We used the meta-analysis of observational studies in epidemiology guidelines in the present study [9].

### Data source and search strategy

Two authors independently performed a literature search of the PubMed, Embase, Cochrane Library, and Chinese public databases, including the China National Knowledge Infrastructure, the China Biology Medicine Database, the China Science Periodical Database (Wanfang Database), and the VIP Journal Integration Platform. The search included studies published through 29 July 2018. The following terms were used in the search strategy: hormone, glucocorticoid, steroid, corticosteroid, osteonecrosis, femoral, femur, femoris, whirlbone, polymorphism, SNP, genetic, mutation, genotype, allele, allelic, and variation. The search strategy is shown in Supplementary Table S1. Divergence in the search results was resolved by discussion.

### Inclusion and exclusion criteria

The studies were included in our meta-analysis if they met the following criteria: (1) case-control or cohort studies comparing a population that suffered SONFH with a population that did not suffer after steroid treatment, (2) studies assessing the associations between genetic polymorphisms and SONFH, and (3) studies reporting the frequencies of specific alleles or the effect sizes of individual genotypes between cases and controls. Studies were excluded from the analysis for the following reasons: (1) noncase-control or noncohort studies, (2) the case group included SONFH patients with other etiologies or SONFH patients who were not reported separately, (3) the control group included an ONFH population without steroid application or a healthy population, (4) non-SNP-related studies, (5) studies about family heredity, and (6) studies that did not report data pertaining to allelic frequencies or effect size. In addition, conference reports, editor comments, reviews, case reports, and academic dissertations were excluded from the analysis.

### Data extraction and quality assessment

Two authors independently extracted the following data from each eligible study: first author’s name, publication year, research location, sample size, average subject age, primary disease, steroid type, cumulative steroid dose, treatment duration, and genes of interest. If the average or median value of the cumulative steroid dosage in each group was not reported, it was estimated. The cumulative dose was generally calculated by multiplying the daily average dose and the treatment duration. For studies with a fixed treatment strategy, the cumulative dosage was calculated by multiplying the dosage of each treatment cycle by the total number of cycles. In the present study, we defined the average body surface area as 1.6 m^2^ and the average body weight as 60 kg. For closed-interval categories, the value assigned to each dosage or duration, such as the average or median, was the midpoint. For open-interval categories, the value was estimated as the limit value multiplied by 1.2 [10]. Because of the different types of steroids used in the included studies, it was necessary to convert the doses of different types of steroids to the equivalent dose of prednisone. The conversion standard is 5 mg prednisone = 5 mg prednisolone = 4 mg methylprednisolone = 0.75 mg dexamethasone [11]. The Newcastle-Ottawa Scale (NOS), a validated tool for evaluating the quality of observation studies, was used to evaluate the methodological quality of the included studies; the scale includes the following three subscales: selection, comparability, and exposure [12].

### Statistical analysis

Association analysis was performed using five genetic models: allelic (W vs M), dominant (WW+WM vs MM), recessive (WW vs WM+MM), heterozygous (WM vs MM), and homozygous (WW vs MM) models [13]. W is the wild allele and M is the mutation allele. The odds ratios (ORs) and their 95% CIs were used to assess the strength of the associations between polymorphisms and SONFH incidence. The I^2^ statistic was used to estimate the degree of heterogeneity amongst the studies. Cochran’s Q statistics were also taken as measures of between-study heterogeneity. Generally, a significance level of *P*<0.1 suggests the existence of significant heterogeneity to expand a test’s sensitivity [13]. If the I^2^ ≥ 50% (Q test, *P*<0.1), the random-effect model was used; otherwise, the fixed-effect model was used. Then, we attempted to use a multilevel mixed-effects logistic regression model, which is an expansion of logistic regression, for each type of steroid, primary disease, drug dose, applied duration, and SNP. The factors of different genotypes and other variables were included as fixed effects, and different studies were considered random effects.

Finally, we conducted a dose-response meta-analysis across the cumulative steroid dosage [14]. To derive the dose-response curve, we modeled the dose using restricted cubic splines with three knots at fixed percentiles of 10, 50, and 90% of the distribution [15]. All tests were two-tailed, and a p-value less than 0.05 was considered statistically significant except in Cochran’s Q test.

## Results

**Figure 1:**
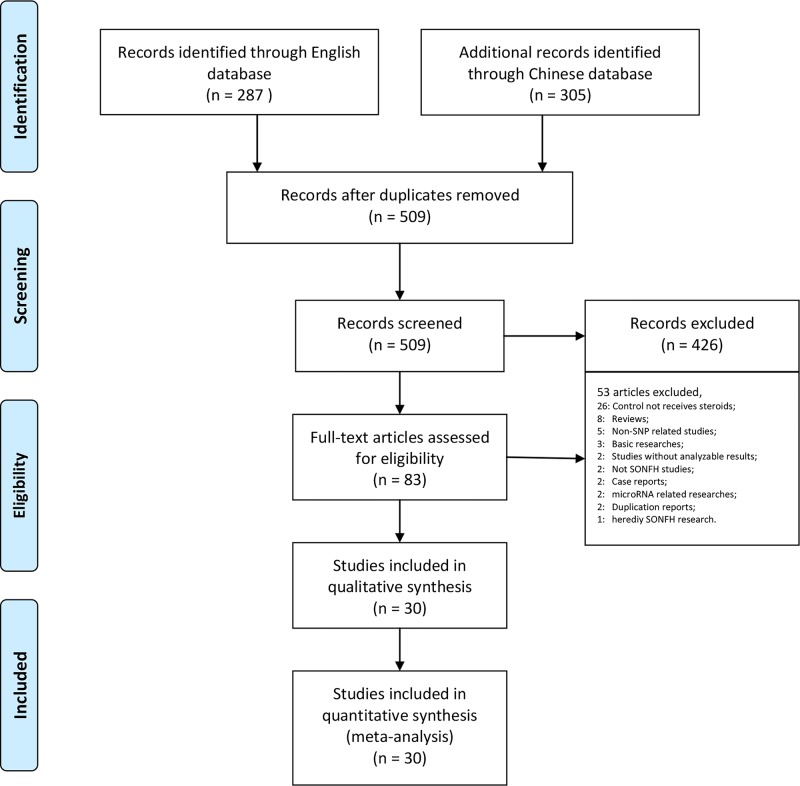
Preferred reporting items for systematic review and meta-analysis flow chart illustrating the process by which the studies included in our analysis were selected

Our research returned 287 English articles and 305 Chinese articles after removing duplicates. After screening the titles and abstracts, 509 of these articles were excluded. The full texts of 83 articles were assessed, amongst which studies were excluded for the following reasons: the control group did not receive steroid therapy (26); reviews (8); non-SNP-related studies (5); basic studies (3); studies without frequencies or effect size results (2); non-steroid-related ONFH studies (2); case reports (2); microRNA-related studies (2); duplicated reports (2); and heredity SONFH study (1). Ultimately, we included 30 studies that assessed a total of 7553 patients in our meta-analysis [16–45] (Figure 1, Table 1).

**Table 1:** Characters of included studies

Author	Year	Local	Sample size	Average age^#^	Type of steroids	Primary disease	Cumulative dose in Exp group	Cumulative dose in Control group	Treatment duration	Genes	NOS score
Zhao et al. [16]	2017	China	193 (78/115)	40 (18–48)	Prednisone	Various	1400 mg	2400 mg	1Y	*GRG*	9
Plesa et al. [17]	2017	Caucasian	304 (32/272)	NA	Prednisone Dexamethasone	ALL	50,400 mg	54,149 mg	120W	*BCL2L11*	9
Karol et al.[18]	2015	Multinational	2955 (400/2555)	NA	Prednisone Dexamethasone	ALL	24,275 mg	24,275 mg	1500D	GWAS	7
X Wei [19]	2015	China	75 (45/30)	39 ± 10	Prednisone	Various	4800 mg	4800 mg	6M	*ApoA1;ApoB; ApoE*	8
Zhang et al. [20]	2014	China	200 (94/106)	44.5 (18–82)	Prednisolone	Various	7300 mg	7300 mg	1Y	*ABCB1*	7
Y Xue [21]	2014	China	322 (105/217)	39 (18–48)	Prednisone	Various	1388 mg	2400 mg	1Y	*ABCB1*	9
Y Cui [22]	2014	China	424 (223/201)	42.27 ± 15.71	Prednisolone	Various	7300 mg	7300 mg	1Y	*ApoA5*	7
P Zeng [23]	2014	China	206 (108/98)	40 ± 10	Prednisone	Various	2400 mg	2400 mg	3M	*ApoB*	8
Zhang et al. [24]	2013	China	200 (94/106)	44.5 (18–82)	Prednisolone	Various	7300 mg	7300 mg	1Y	*PAI–1*	7
Wang et al.[25]	2013	China	200 (94/106)	44.5 (18–82)	Prednisolone	Various	7300 mg	7300 mg	1Y	*PON-1*	6
Y Li [26]	2012	China	123 (70/53)	29 (18–73)	Prednisone	Various	480 mg	480 mg	6M	*ABCB1*	6
W Fang [27]	2011	China	134 (63/71)	35.17 ± 11.73	Prednisone	Various	6991 mg	5014 mg	6M	*ApoB; CYP1A2*	8
W Fang [28]	2011	China	134 (63/71)	35.17 ± 11.73	Prednisone	Various	6991 mg	5014 mg	6M	*Factor V;GR;CBP; ApoB;CYP1A2*	8
J Bond [29]	2011	U.K.	110 (43/67)	NA	Dexamethasone	ALL	68,333 mg	68,333 mg	143W	*PAI-1*	8
W He [30]	2009	China	48 (31/17)	32 (12–59)	Prednisone	Hemoglobinopathies	480 mg	480 mg	2M	*CYP3A4/ ABCB1*	7
W He [31]	2009	China	48 (31/17)	18–60	Prednisone	Various	480 mg	480 mg	6M	*CYP3A4*	7
Kuribayashi et al. [32]	2008	Japan	157 (34/123)	35 (9–64)	Methylprednisolone Prednisolone	RT	3227 mg	3207 mg	6M	*ABCB1; ApoB; CBP*	9
D French [33]	2008	U.S.A.	361 (51/310)	NA (10–20)	Prednisone Dexamethasone	ALL	15,139 mg	15,139 mg	NA	*ABCB1;PAI–1 et al. 11 Genes*^##^	7
Wang et al.[34]	2008	China	53 (16/37)	35 (16–78)	Methylprednisolone	SARS	5672 mg	4187 mg	NA	*TNF-a*	8
Tamura et al. [35]	2007	Japan	157 (34/123)	35 (9–64)	Methylprednisolone Prednisolone	RT	3251 mg	3215 mg	6M	*GR;CYP3A4; CBP;NCoA2*	9
XY Yang [36]	2007	China	127 (21/106)	34 (11–67)	Methylprednisolone Prednisone	SLE	89,173 mg	89,173 mg	5Y	*ABCB1*	9
Hirata et al.[37]	2007	Japan	112 (20/92)	NA	Methylprednisolone Prednisone	RT	3223 mg	3223 mg	6M	*ApoA*	7
Hirata et al.[38]	2007	Japan	158 (34/124)	36.1 (9–64)	Methylprednisolone Prednisolone	RT	3223 mg	3223 mg	6M	*ApoB*	7
Ekmekci et al. [39]	2006	Turkey	57 (19/38)	34.2 ± 9.3	NA	RT	NA	NA	20.6M	*Factor V, Prothrombin*	7
Celik et al.[40]	2006	Turkey	50 (11/39)	41 ± 11.79	Prednisolone	RT	8835 mg	6322 mg	18M	*Factor V, Prothrombin, MTHFR*	8
Relling et al. [41]	2004	U.S.A.	64 (25/39)	8.6 (2.7–18.8)	Prednisone	ALL	17,028 mg	17,867 mg	1.23Y	*MDR1(ABCB1) et al. 13 Genes*^###^	8
Asano et al. [42]	2004	Japan	137 (31/106)	36 (9–63)	Methylprednisolone Prednisolone	RT	3228 mg	3174 mg	NA	*PAI-1; MTHFR*	8
Asano et al. [43]	2003	Japan	80 (26/54)	NA	NA	RT	NA	NA	2Y	*CYP3A4; CYP2D6; CYP2C19*	7
Asano et al. [44]	2003	Japan	136 (30/106)	35.5 (9–63)	Methylprednisolone Prednisolone	RT	3227 mg	3207 mg	6M	*ABCB1*	8
Ferrari et al. [45]	2002	Switzerland	228 (26/202)	50 ± 12	Prednisone	RT	9600 mg	9600 mg	8Y	*PAI-1*	8

ALL, acute lymphoblastic leukemia; GWAS, genome-wide association study; NA, not available; RT, renal transplant; SARS, severe acute respiratory syndrome.

# Mean ± standardization; mean/median (minimum–maximum).

## TYMS; VDR; BGLAP; ESR1; LRP5; MTHFR; PAI-1; ABCB1(MDR1); PTH; PTHR; ACP5.

### CYP3A4; CYP3A5; TPMT; UGT1A1; TYMS; GSTT1; GSTM1; RFC; MTHFR; GRG(NR3C1); MDR1(ABCB1); VDR; GSTP1.

All studies were case-control designs, except one study, which was a cohort design [18]. The primary diseases included organ transplantation, hematological disease, systemic lupus erythematosus (SLE), rheumatoid arthritis (RA), nephrotic syndrome, and ophthalmic disease. In addition, two studies by Wei He [30,31], two studies by Weibao Fang [27,28], and studies by Masaaki and Kyoko [32,35] included the same patient groups but did not assess the same SNPs. The NOS scores ranged from six to nine points, and the overall quality of the observational studies was ideal (Table 1). The details of NOS scores for each study are shown in Supplementary Table S2.

**Figure 2:**
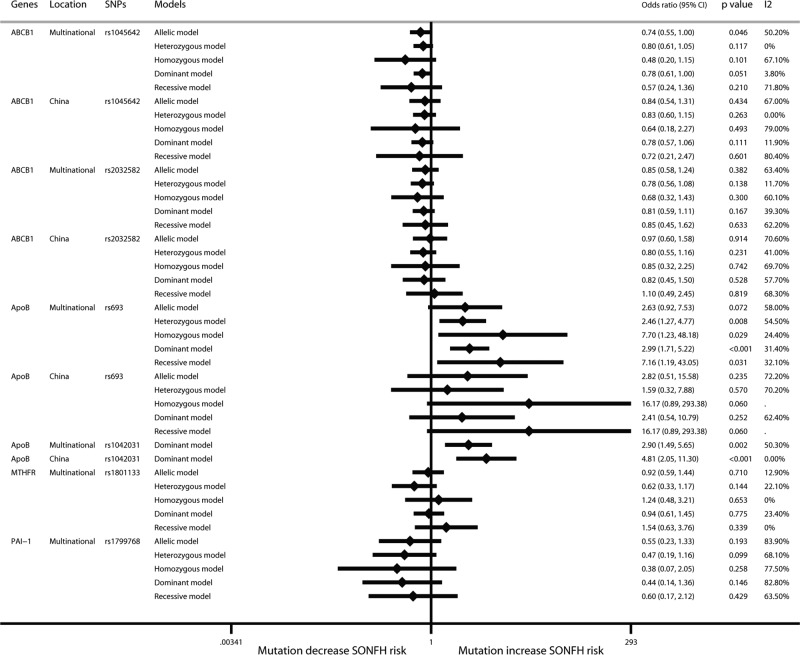
Forest plot of SONFH risk of SNP carriers that are included in more than three studies by traditional meta-analysis

Amongst the preliminary results of SNPs that were included in more than three studies, an allelic model showed that the ABCB1 rs1045642 mutation had a protective effect against SONFH (OR: 0.74; 95% CI: 0.55–1.00; *P*=0.046). ApoB rs693 (heterozygous model: OR: 2.46; 95% CI: 1.27–4.77; *P*=0.008; homozygous model: OR: 7.70; 95% CI: 1.23–48.16; *P*=0.029; dominant model: OR: 2.99; 95% CI: 1.71–5.21; *P*<0.001; recessive model: OR: 7.16; 95% CI: 1.19–43.02; *P*=0.031) and rs1042031 (dominant model: OR: 2.90; 95% CI: 1.49–5.66; *P*=0.002) mutations increased the risk of SONFH. ABCB1 rs2032582, MTHFR rs1801133, and PAI-1 rs1799768 had no significant relationships with SONFH (Figure 2). Other SNPs were not included in the analysis because they did not appear in more than three studies. A further analysis was performed in the present study using a multilevel mixed-effects logistic regression model. The details of the included studies for each group analysis are listed in Supplementary Table S3.

**Figure 3:**
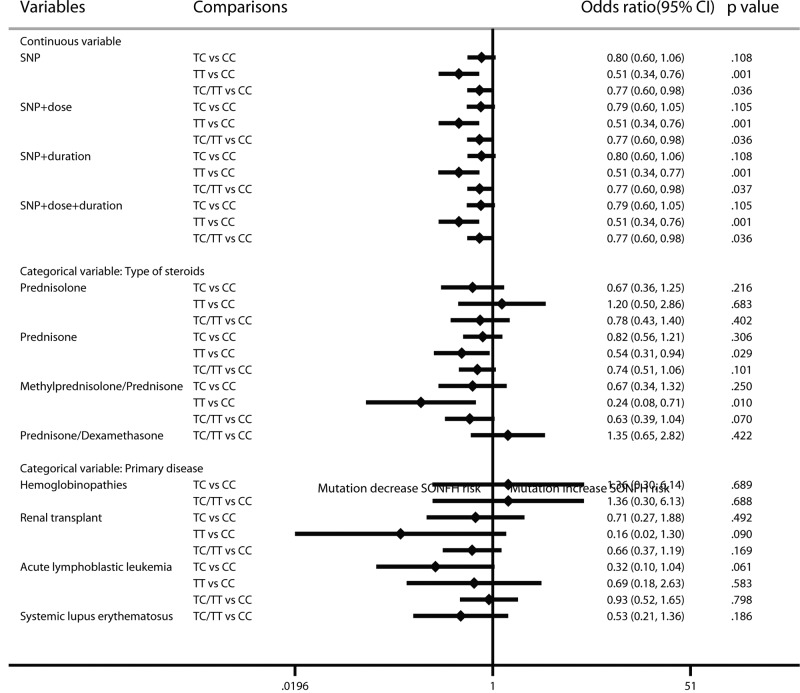
Forest plot of ABCB1 rs1045642 mutation on SONFH risk by the multilevel mixed-effects logistic regression model

In the results of the ABCB1 rs1045642 multilevel mixed-effects regression model, the mutant homozygote and the mutant carrier could significantly protect against SONFH after steroid application (TT vs CC genotype: OR: 0.51; 95% CI: 0.34–0.76; *P*=0.001; TC/TT vs CC: OR: 0.77; 95% CI: 0.60–0.98; *P*=0.036). The results showed no significant changes in the cumulative dosage and treatment duration. According to the analyses of categorical variables of steroid type, in the prednisone-use population, mutant homozygotes had a significant protective effect compared with the wild-type population (TT vs CC: OR: 0.54; 95% CI: 0.31–0.94; *P*=0.029), and the results were similar in the methylprednisolone/prednisone-use population (TT vs CC: OR: 0.24; 95% CI: 0.08–0.71; *P*=0.010). The analysis of primary disease showed no significant results. It should be noted that the studies that did not report primary disease type or that included several types of primary disease were not included in the categorical analysis of primary disease (Figure 3).

**Figure 4:**
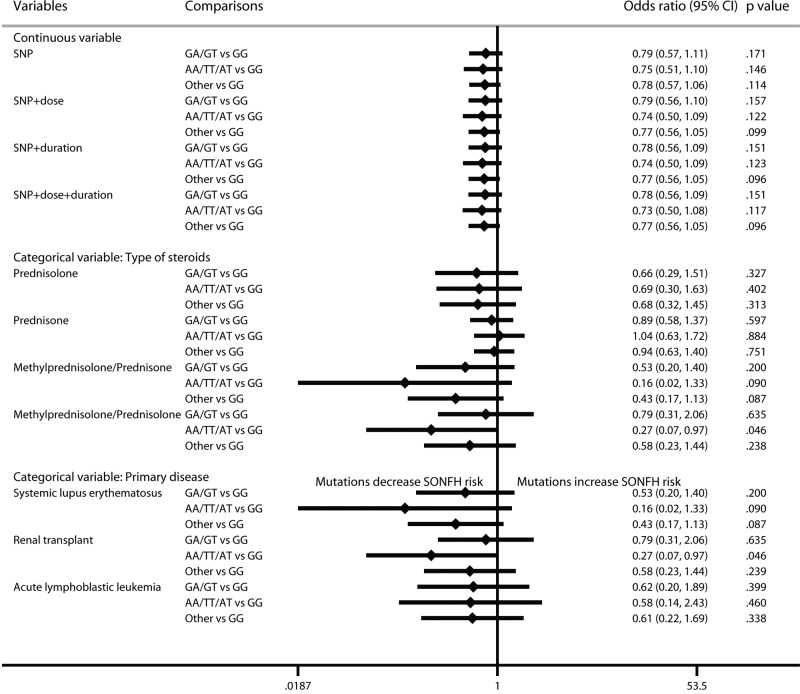
Forest plot of ABCB1 rs2032582 mutation on SONFH risk by the multilevel mixed-effects logistic regression model

For ABCB1 rs2032582, the negative results did not change after considering cumulative dose and treatment duration. In the categorical analysis, the protective effect of the mutant was only found in the methylprednisolone/prednisolone-use renal transplant population (OR: 0.27; 95% CI: 0.07–0.97; *P*=0.046). However, this result was based on a single study and had a large standard error (SE = 0.66); thus, this result needs to be confirmed (Figure 4).

**Figure 5:**
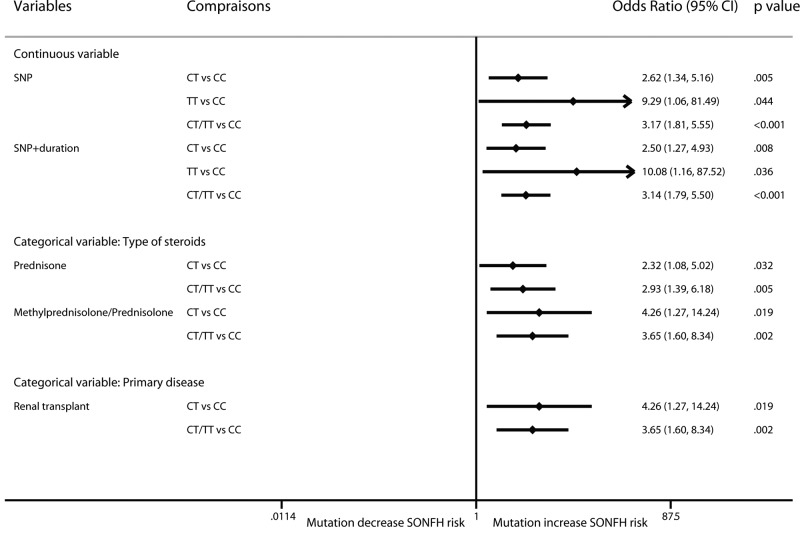
Forest plot of ApoB rs693 mutation on SONFH risk by the multilevel mixed-effects logistic regression model

For ApoB rs693, mutation increased the incidence of SONFH (CT vs TT: OR: 2.62; 95% CI: 1.34–5.16; *P*=0.005; TT vs CC: OR: 9.29; 95% CI: 1.06–81.49; *P*=0.044; CT/TT vs CC: OR: 3.16; 95% CI: 1.81–5.55; *P*<0.001) (Figure 5). Due to a large number of zero events, the regression results did not converge after considering the cumulative dose. The results were not changed after considering treatment duration. In addition, in prednisone-use and methylprednisolone/prednisolone-use populations and renal transplant patients, mutation increased the risk of SONFH.

**Figure 6:**
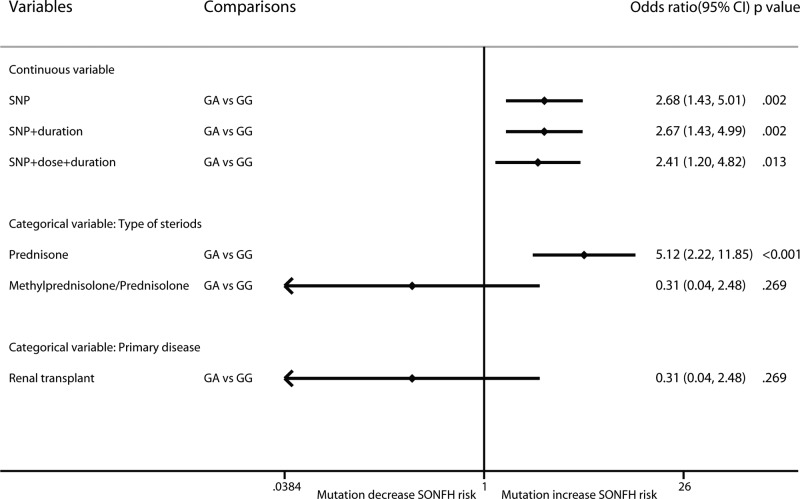
Forest plot of ApoB rs1042031 mutation on SONFH risk by the multilevel mixed-effects logistic regression model

For ApoB rs1042031, mutation increased the risk of SONFH (GA vs GG: OR: 2.68; 95% CI: 1.43–5.01; *P*=0.002) (Figure 6). The results were not obviously changed after considering cumulative dose and treatment duration. In the categorical analysis, mutation increased the risk of SONFH in the prednisone-use population (OR: 5.12; 95% CI: 2.22–11.85; *P*<0.001), but not in the methylprednisolone/prednisolone-use renal transplant population (OR: 0.31; 95% CI: 0.04–2.48; *P*=0.269).

**Figure 7:**
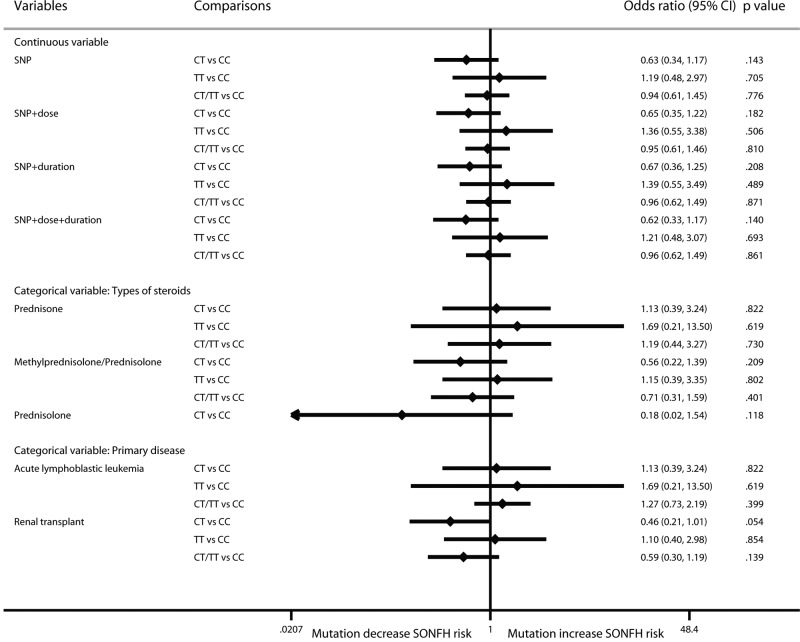
Forest plot of MTHFR rs1801133 mutation on SONFH risk by the multilevel mixed-effects logistic regression model

For MTHFR rs1801133, there was no significant correlation between mutation and SONFH risk (CT vs CC: OR: 0.63; 95% CI: 0.34–1.17; *P*=0.143; TT vs CC: OR: 1.19; 95% CI: 0.48–2.97; *P*=0.705; CT/TT vs CC: OR: 0.94; 95% CI: 0.61–1.45; *P*=0.776). The results did not change after considering the continuous and categorical variables (Figure 7).

**Figure 8:**
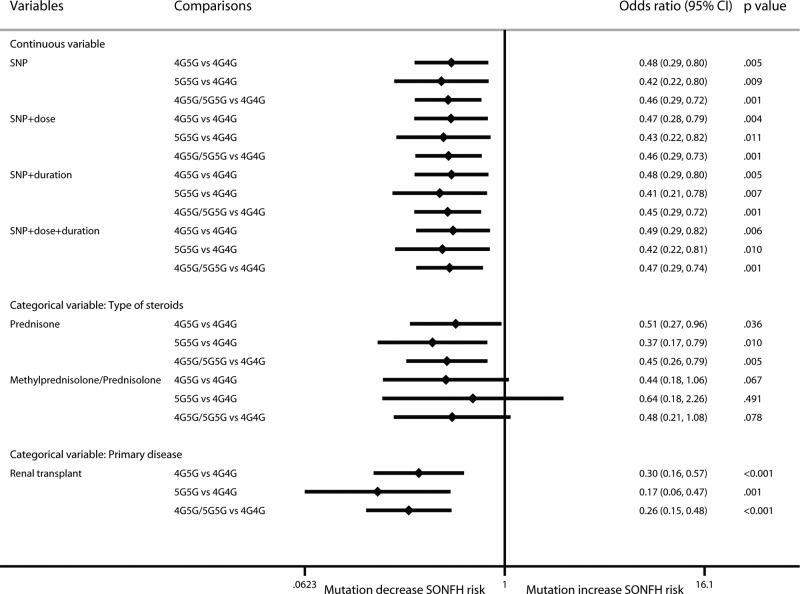
Forest plot of PAI-1 rs1799768 mutation on SONFH risk by the multilevel mixed-effects logistic regression model

For PAI-1 rs1799768, 5G mutation had a protective effect against SONFH (4G5G vs 5G5G: OR: 0.48; 95% CI: 0.29–0.80; *P*=0.005; 5G5G vs 4G4G: OR: 0.42; 95% CI: 0.22–0.80; *P*=0.009; 4G5G/5G5G vs 4G4G: OR: 0.46; 95% CI: 0.29–0.72; *P*=0.001) (Figure 8). These results differed from previous results because in previous research a random-effect model was used due to large heterogeneity, and a mixed-effect model was adopted in this research. The results did not change after considering cumulative dose and treatment duration. In the categorical analysis, the protective effect existed in the prednisone-use and renal transplant population. However, there was no significant correlation in the methylprednisolone/prednisolone-use population (4G5G vs 5G5G: OR: 0.44; 95% CI: 0.18–1.06; *P*=0.067; 5G5G vs 4G4G: OR: 0.64; 95% CI: 0.18–2.26; *P*=0.491; 4G5G/5G5G vs 4G4G: OR: 0.48; 95% CI: 0.21–1.08; *P*=0.078).

**Figure 9:**
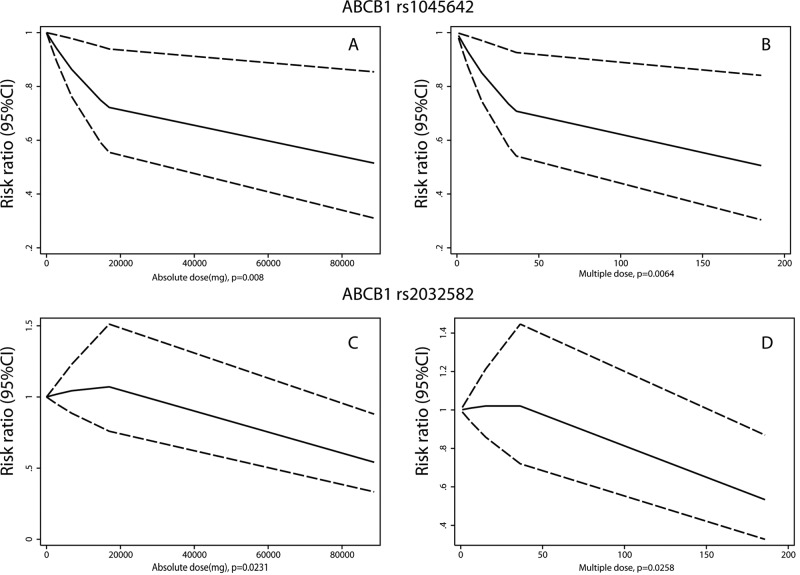
Dose-response analyses of cumulative steroid dose and SONFH risk in mutation carriers (**A**) Analysis of absolute dose in ABCB1 rs1045642 mutation carriers; (**B**) analysis of multiple dose in ABCB1 rs1045642 mutation carriers; (**C**) analysis of absolute dose in ABCB1 rs2032582 mutation carriers; (**D**) analysis of multiple dose in ABCB1 rs2032582 mutation carriers.

Furthermore, we sought to analyze the cumulative dosage and SONFH risk in mutation carriers by a dose-response meta-analysis. SONFN/ONFH incidence in mutation carriers cannot be obtained when steroids are not used. Therefore, the minimum dose must be used as a reference, and whether increasing the dose affects SONFH incidence must be assessed. Two dose assessment methods were used: an absolute dose and multiple doses. For ABCB1 rs1045642, there was a correlation between the cumulative dose and the occurrence of SONFH in the mutation carriers. As the dose increased, the risk of SONFH decreased (absolute dose: *P*=0.008; multiple dose: *P*=0.0064) (Figure 9A,B). This negative correction also persists in homozygous mutation (TT genotype, absolute dose: *P*=0.0368; multiple dose: *P*=0.0435) and heterozygous mutation patients (CT genotype, absolute dose: *P*<0.001; multiple dose: *P*<0.001) (Supplementary Figure S1A–D). In wild-type populations, there was no correlation between the dose and the risk of SONFH (absolute dose: *P*=0.098; multiple dose: *P*=0.098). Although there was no significant correlation between the ABCB1 rs2032582 mutation and the risk of SONFH, the cumulative dose in the mutant population was correlated with the occurrence of SONFH. As the dose increased, the risk of SONFH decreased (absolute dose: *P*=0.0231; multiple dose: *P*=0.0258) (Figure 9C,D). This negative correction mainly persists in heterozygous mutation patients (GA/GT genotype, absolute dose: *P*=0.0286; multiple dose: *P*=0.0293), but not in other mutation patients (AA/AT/TT genotype, absolute dose: *P*=0.7103; multiple dose: *P*=0.7716) (Supplementary Figure S1E–H). In wild-type populations, there was no correlation between the dose and the risk of SONFH (absolute dose: *P*=0.2821; multiple dose: *P*=0.285). These findings may suggest that mutation carriers still have a risk of SONFH when using steroids, similar to the wild-type population. If SONFH does not occur after small-dose steroid application, increasing the steroid dose in mutation carriers will not increase but rather decrease the risk of SONFH.

For ApoB rs693, although SONFH was correlated with the dose in the mutation carriers (absolute dose: *P*=0.0064; multiple dose: *P*=0.0064), a correlation was also found in the wild-type population (absolute dose: *P*=0.0177; multiple dose: *P*=0.0013). For ApoB rs1042031, there was no correlation between the cumulative dose and SONFH risk in the mutation carriers (absolute dose: *P*=0.662; multiple dose: *P*=0.7078) or in the wild-type population (absolute dose: *P*=0.3106; multiple dose: *P*=0.0719). ApoB rs693 and rs1042031 have too few homozygous mutation patients to analyze separately. We did not perform a dose-response analysis for MTHFR rs1801133 and PAI-1 rs1799768 due to the small number of included studies.

## Discussion

ABCB1 rs1045642 mutations have a protective effect against SONFH, and mutations in ApoB rs693 and rs1042031 increase the SONFH risk. The present study further analyzed the effects of each specific characteristic, such as the primary disease of the included patients, the types of steroids, the cumulative dosage, and the treatment duration, on outcomes. However, with the increase in variable factors, the results will be more dependent on the results of single studies, which will reduce the stability of the results. However, the analyses performed here can provide additional clues and research directions for further basic and clinical research studies. In previous research, we analyzed the associations between SNPs and SONFH by traditional meta-analysis, and more than three SNPs were included. Ultimately, ABCB1 rs1045642, rs2032582, ApoB rs693, rs1042031, MTHFR rs1801133, and PAI-1 rs1799768 were included.

In previous meta-analyses, one study analyzed the association between ABCB1 gene polymorphism and SONFH and the 3435T (rs1045642) and 2677T/A (rs2032582) alleles of ABCB1 were identified, which may reduce the risk of GC-induced ONFH [6]. However, our study included more reports and found that rs2032582 had no correlation with SONFH. Our result is the same as that from another meta-analysis that analyzed the correlation between gene polymorphism and SONFH occurrence with the GRADE method to assess the level of evidence. The results showed that ABCB1 rs1045642 could reduce the occurrence of SONFH but had a very low level of evidence. ApoB rs693 and rs1042031 mutations increased the risk of SONFH and had a moderate level of evidence [46]. The results of our study are essentially the same as those of the above two studies, except for rs2032582. However, our work further analyzed the associations between SNP and SONFH based on steroid type, primary disease, drug dose, and applied duration using a mixed-effects logistic regression model. We also conducted a dose-response meta-analysis to analyze the cumulative dosage and SONFH risk in mutation carriers. Our work expanded the research results and found that cumulative steroid dosage and treatment duration had little effect on the results. There was a dose-effect correlation in ABCB1 rs1045642 and rs2032582 mutation carriers. Another study analyzed the associations between ONFH and genetic polymorphisms of vascular endothelial growth factor rs2010963, endothelial nitric oxide synthase rs2070744, and ABCB1 rs1045642. However, the included studies contained SONFH- and ONFH-related studies. The results showed that the mutations of rs2010963 and rs1045642 increased the risk of ONFH, and the mutation of rs2070744 increased the risk of ONFH in an allelic model. There were errors in the present study, as the authors proposed that the mutation of ABCB1 rs1045642 increased the risk of ONFH, but the data showed that it reduced the risk [47]. Our study analyzed SONFH risk, but not ONFH, which is different from the above study.

In the present study, there were significant correlations between the ABCB1 rs1045642 mutant and SONFH in the prednisone-use and methylprednisolone/prednisone-use populations. The ABCB1 rs2032582 mutant homozygote had a protective effect in the methylprednisolone/prednisolone renal transplant population. For ApoB rs693, mutation increased the incidence of SONFH in the prednisone-use and methylprednisolone/prednisolone-use populations and renal transplant patients. For ApoB rs1042031, mutation increased the risk of SONFH in the prednisone-use population. The PAI-1 rs1799768 mutation had a protective effect in the SONFH risk prednisone-use and renal transplant population.

There were different outcomes for the different types of steroid-use populations, possibly because the results were based on even fewer studies. These differences may also indicate that different types of steroids have subtle differences in affecting the risk of SONFH. It is generally believed that prednisone must be transformed to an active form through the liver, while prednisolone does not require this change, which may be the reason for the different results of the two drugs in ABCB1-related mutation states. In addition, more evidence is needed to determine whether the different results for the types of steroids in ApoB and PAI-1 gene polymorphisms were due to different effects on adipocytes.

Currently, a registered study is analyzing the pharmacokinetics of different steroids in children, but the results have not yet been reported (NCT02252237). An earlier study also reported that the cumulative dosage of methylprednisolone, but not prednisone, is correlated with avascular necrosis incidence [48]. Therefore, defined steroid types should be evaluated in further genetic polymorphism- and SONFH-related studies.

Although there are reports about the effect of primary disease on SONFH risk, most have focussed on the influence of steroids used in disease treatment. It was also reported that in renal transplant patients, cyclosporine versus tacrolimus as well as gender factors may be independent risk factors for avascular necrosis [49]. A meta-analysis of the avascular necrosis risk in SLE patients also showed, that in addition to hormonotherapy, arthritic, cushingoid, hypertension, cytotoxic drugs, etc., also affect the occurrence of SONFH [50]. Therefore, the primary disease and the treatment strategy are also possible risk factors for SONFH incidence.

It is generally believed that the occurrence of SONFH is due to long-term and/or high-dosage steroid application. A dose-response meta-analysis showed that the incidence of osteonecrosis is correlated with both the cumulative dose and treatment duration [51]. However, there is still no related research on specific mutant carriers. First, the present study found that the ABCB1 rs1045642 mutation has a protective effect on SONFH, and the risk will be further reduced with increasing cumulative steroid dosage. It can also be concluded that some mutant carriers will have SONFH when the cumulative dosage is low; however, if SONFH does not occur, the risk of SONFH will not increase when the cumulative dose of steroids is increased. Therefore, for mutant carriers, the critical time period for prevention and diagnosis of SONFH is during the early stage of steroid application, not the late stage. A negative correlation was also found in ABCB1 rs2032582 mutation carriers, even though the mutations were not significantly associated with SONFH in general.

## Limitation

The present study still has several limitations. First, our study was performed at the study level instead of the individual level. Second, in stratified analyses, with the increase in variable factors, the results become more dependent on the results of single studies, which will reduce the stability of the results. Third, in dose-response analyses, SONFN/ONFH incidence in mutation carriers without steroid application cannot be calculated; therefore, the minimum dose must be used as a reference. Fourth, our study only analyzed SNPs that were examined in more than three studies, but with emerging research and evidence, more SNPs may be studied in the future. Therefore, the present study was limited by the research available at the time.

## Supporting information

**Supplementary Figure S1:** 

**Supplementary Table S1:** Search queries in each database.

**Supplementary Table S2:** NOS score details for each included study.

**Supplementary Table S3:** Details of the included studies for the analysis of each group in the meta-analysis.

## Abbreviations

NOS: Newcastle-Ottawa Scale
OR: odds ratio
RA: rheumatoid arthritis
SLE: systemic lupus erythematosus
SNP: single-nucleotide polymorphism
SONFH: steroid-induced osteonecrosis of the femoral head
