# Supplementary material for: Association between genetic polymorphisms and osteonecrosis in steroid treatment populations: a detailed stratified and dose-response meta-analysis
Source: Biosci Rep. 2019 May 14;39(5):BSR20190024. doi: 10.1042/BSR20190024 (PMC6522878; doi:10.1042/BSR20190024)
Supplement: Supplementary file 1 [file BSR-2019-0024_suppS1.pdf]

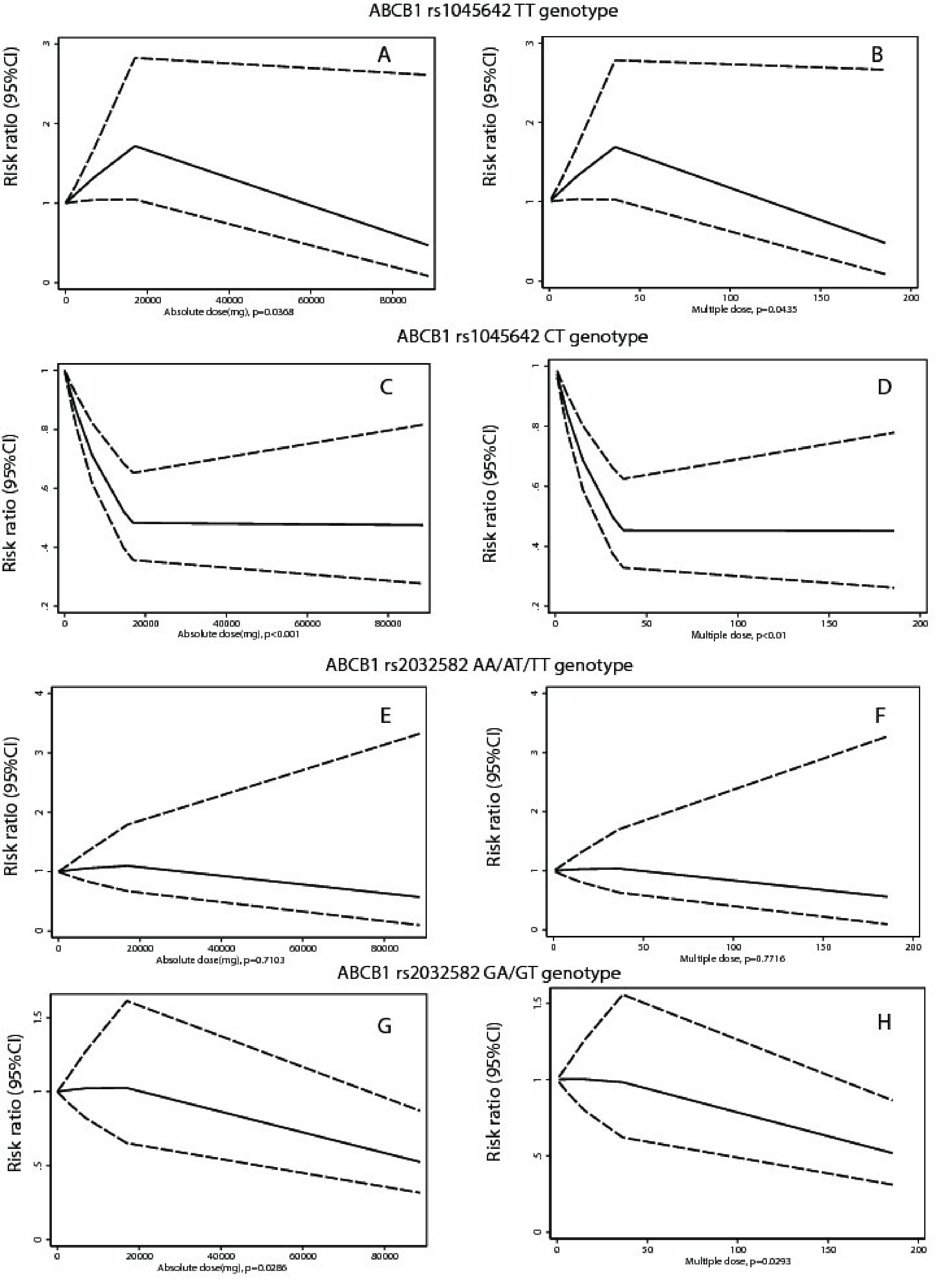
ABCB1 rs1045642 TT genotype
A
B
Risk ratio (95%CI)
Absolute dose(mg), p=0.0368
Multiple dose, p=0.0435
ABCB1 rs1045642 CT genotype
C
D
Risk ratio (95%CI)
Absolute dose(mg), p<0.001
Multiple dose, p<0.01
ABCB1 rs2032582 AA/AT/TT genotype
E
F
Risk ratio (95%CI)
Absolute dose(mg), p=0.7103
Multiple dose, p=0.7716
ABCB1 rs2032582 GA/GT genotype
G
H
Risk ratio (95%CI)
Absolute dose(mg), p=0.0286
Multiple dose, p=0.0293

Supplementary table 1. Search queries in each database.

PubMed:

Search	Query	Items found	Time
#5	Search (((#1) AND #2) AND #3) AND #4	108	4:38:46
#4	Search (polymorphism or polymorphisms or SNP or genetic or mutation or genotype or allele or allelic or variation)	2522483	4:38:13
#3	Search (femoral OR femur OR femoris OR whirlbone)	192476	4:38:06
#2	Search (Osteonecrosis OR necrosis)	363077	4:37:57
#1	Search (hormone OR glucocorticoid OR steroid OR corticosteroid)	1865321	4:37:47

EmBase:

Search	Query	Items found
#25	#21 AND #22 AND #23 AND #24	202
#24	#11 OR #12 OR #13 OR #14 OR #15 OR #16 OR #17 OR #18 OR #19 OR #20	2,983,625
#23	#7 OR #8 OR #9 OR #10	259,727
#22	#5 OR #6	593,833
#21	#1 OR #2 OR #3 OR #4	1,286,683
#20	variation	589,499
#19	allelic	50,116
#18	allele	257,725
#17	genotype	399,242
#16	mutation	838,980
#15	genetic	1,812,465
#14	snps	71,321
#13	snp	75,330
#12	polymorphisms	165,377
#11	polymorphism	404,079
#10	whirlbone	4
#9	femoris	22,759
#8	femur	105,777
#7	femoral	185,332
#6	necrosis	589,024
#5	osteonecrosis	13,356
#4	'corticosteroid'	274,435
#3	'steroid'	316,874
#2	glucocorticoid	112,839
#1	'hormone'/exp OR hormone	735,482

Cochrane Library:

Search	Query	Items found

#1	hormone OR glucocorticoid OR steroid OR corticosteroid	53248
#2	Osteonecrosis OR necrosis	13895
#3	femoral OR femur OR femoris OR whirlbone	12510
#4	polymorphism or polymorphisms or SNP or genetic or mutation or genotype or allele or allelic or variation	48102
#5	#1 and #2 and #3 and #4	42

Chinese search strategy:
(激素性骨坏死 OR 激素性股骨头坏死) and (基因多态性 or 多态性 or 多态性)
China National Knowledge Infrastructure (CNKI): 47 items

the China Biology Medicine Database: 17 items

the China Science Periodical Database (CSPD, Wanfang Database): 282 items

the VIP Journal Integration Platform (VJIP): 23 items

Supplementary table 2. NOS score details for each included study.

Author	NOS score	Selection				Comparability	Exposure		
		Is the case definition adequate?	Representativeness of the case	Selection of controls	Definition of controls	Comparability of cases and controls on the basis of the design or analysis	Ascertainment of exposure	Same method of ascertainment for cases and controls	Non-response rate
Zhanqin Zhao 2017[16]	9	Yes	Yes	Yes	Yes	Yes	Yes	Yes	Yes
Maria Plesa 2017[17]	9	Yes	Yes	Yes	Yes	Yes	Yes	Yes	Yes
Seth E.Karol 2015[18]	7	Yes	Yes	No description or inappropriate	Yes	Steroid-related confounding factors were not controlled	Yes	Yes	Yes
Xiangdong Wei 2015[19]	8	No description or inappropriate	Yes	Yes	Yes	Yes	Yes	Yes	Yes
Yanqiong Zhang 2014[20]	7	No description or inappropriate	Yes	No description or inappropriate	Yes	Yes	Yes	Yes	Yes
Yun Xue 2014[21]	9	Yes	Yes	Yes	Yes	Yes	Yes	Yes	Yes
Yong Cui 2014[22]	7	Yes	Yes	No description or inappropriate	Yes	Steroid-related confounding factors were not controlled	Yes	Yes	Yes
Ping Zeng 2014[23]	8	No description or inappropriate	Yes	Yes	Yes	Yes	Yes	Yes	Yes
Yanqiong Zhang 2013[24]	7	No description or inappropriate	Yes	No description or inappropriate	Yes	Yes	Yes	Yes	Yes
Zhiyao Wang 2013[25]	6	No description or inappropriate	Yes	No description or inappropriate	Yes	Yes	Yes	Yes	Rate different
Yong Li 2012[26]	6	No description or inappropriate	Yes	No description or inappropriate	Yes	Steroid-related confounding factors were not controlled	Yes	Yes	Yes
Weibiao Fang 2011[27]	8	Yes	Yes	Yes	No description or inappropriate	Yes	Yes	Yes	Yes
Weibiao Fang 2011[28]	8	Yes	Yes	No description or inappropriate	Yes	Yes	Yes	Yes	Yes
Jonathan Bond 2011[29]	8	No description or inappropriate	Yes	Yes	Yes	Yes	Yes	Yes	Yes
Wei He 2009[30]	7	Yes	Yes	Yes	No description or inappropriate	Steroid-related confounding factors were not controlled	Yes	Yes	Yes
Wei He 2009[31]	7	Yes	Yes	No description or inappropriate	Yes	Steroid-related confounding factors were not controlled	Yes	Yes	Yes
Masaaki Kuribayashi 2008[32]	9	Yes	Yes	Yes	Yes	Yes	Yes	Yes	Yes
Deborah French 2008[33]	7	Yes	Yes	No description or inappropriate	No description or inappropriate	Yes	Yes	Yes	Yes
Shixin Wang 2008[34]	8	Yes	Yes	No description or inappropriate	Yes	Yes	Yes	Yes	Yes
Kyoko Tamura 2007[35]	9	Yes	Yes	Yes	Yes	Yes	Yes	Yes	Yes
X.Y Yang 2007[36]	9	Yes	Yes	Yes	Yes	Yes	Yes	Yes	Yes
Tetsurou Hirata 2007[37]	7	No description or inappropriate	Yes	No description or inappropriate	Yes	Yes	Yes	Yes	Yes
Tetsurou Hirata 2007[38]	7	No description or inappropriate	Yes	No description or inappropriate	Yes	Yes	Yes	Yes	Yes
Yakup Ekmekci 2006[39]	7	Yes	Yes	Yes	No description or inappropriate	Steroid-related confounding factors were not controlled	Yes	Yes	Yes
A.Celik 2006[40]	8	Yes	Yes	No description or inappropriate	Yes	Yes	Yes	Yes	Yes
Mary V.Relling 2004[41]	8	Yes	Yes	No description or inappropriate	Yes	Yes	Yes	Yes	Yes
Takeshi Asano 2004[42]	8	Yes	Yes	No description or inappropriate	Yes	Yes	Yes	Yes	Yes
Takeshi Asano 2003[43]	7	Yes	Yes	No description or inappropriate	Yes	Steroid-related confounding factors were not controlled	Yes	Yes	Yes
Takeshi Asano 2003[44]	8	Yes	Yes	No description or inappropriate	Yes	Yes	Yes	Yes	Yes
Paolo Ferrari 2002[45]	8	Yes	Yes	Yes	No description or inappropriate	Yes	Yes	Yes	Yes

Supplementary Table 3. Details of the included studies for the analysis of each group in the meta-analysis.

Gene	Location	SNP	Model/Category	Subgroups	No. of studies	References
ABCB1	Multinational	rs1045642	Allelic model		8	Yanqiong Zhang 2014[20];Yun Xue 2014[21];Wei He 2009[30];Masaaki Kuribayashi 2008[32];X.Y Yang 2007[36];Takeshi Asano 2003[44];Yong Li 2012[26];Mary V.Relling 2004[41]
			Heterozygous model		8	Yanqiong Zhang 2014[20];Yun Xue 2014[21];Wei He 2009[30];Masaaki Kuribayashi 2008[32];X.Y Yang 2007[36];Takeshi Asano 2003[44];Yong Li 2012[26];Mary V.Relling 2004[41]
			Homozygous model		8	Yanqiong Zhang 2014[20];Yun Xue 2014[21];Wei He 2009[30];Masaaki Kuribayashi 2008[32];X.Y Yang 2007[36];Takeshi Asano 2003[44];Yong Li 2012[26];Mary V.Relling 2004[41]
			Dominant model		9	Yanqiong Zhang 2014[20];Yun Xue 2014[21];Wei He 2009[30];Masaaki Kuribayashi 2008[32];X.Y Yang 2007[36];Takeshi Asano 2003[44];Yong Li 2012[26];Mary V.Relling 2004[41];Deborah French 2008[33]
			Recessive model		8	Yanqiong Zhang 2014[20];Yun Xue 2014[21];Wei He 2009[30];Masaaki Kuribayashi 2008[32];X.Y Yang 2007[36];Takeshi Asano 2003[44];Yong Li 2012[26];Mary V.Relling 2004[41]
ABCB1	China	rs1045642	Allelic model		5	Yanqiong Zhang 2014[20];Yun Xue 2014[21];Wei He 2009[30];X.Y Yang 2007[36];Yong Li 2012[26]
			Heterozygous model		5	Yanqiong Zhang 2014[20];Yun Xue 2014[21];Wei He 2009[30];X.Y Yang 2007[36];Yong Li 2012[26]
			Homozygous model		5	Yanqiong Zhang 2014[20];Yun Xue 2014[21];Wei He 2009[30];X.Y Yang 2007[36];Yong Li 2012[26]
			Dominant model		5	Yanqiong Zhang 2014[20];Yun Xue 2014[21];Wei He 2009[30];X.Y Yang 2007[36];Yong Li 2012[26]
			Recessive model		5	Yanqiong Zhang 2014[20];Yun Xue 2014[21];Wei He 2009[30];X.Y Yang 2007[36];Yong Li 2012[26]
		rs1045642	Continuous variable			
				TC vs CC	8	Yanqiong Zhang 2014[20];Yun Xue 2014[21];Wei He 2009[30];Masaaki Kuribayashi 2008[32];X.Y Yang 2007[36];Takeshi Asano 2003[44];Yong Li 2012[26];Mary V.Relling 2004[41]
				TT vs CC	8	Yanqiong Zhang 2014[20];Yun Xue 2014[21];Wei He 2009[30];Masaaki Kuribayashi 2008[32];X.Y Yang 2007[36];Takeshi Asano 2003[44];Yong Li 2012[26];Mary V.Relling 2004[41]
				TC/TT vs CC	9	Yanqiong Zhang 2014[20];Yun Xue 2014[21];Wei He 2009[30];Masaaki Kuribayashi 2008[32];X.Y Yang 2007[36];Takeshi Asano 2003[44];Yong Li 2012[26];Mary V.Relling 2004[41];Deborah French 2008[33]
			Type of steroids			

				Prednisolone	1	Yanqiong Zhang 2014[20]
				Prednisone	4	Yun Xue 2014[21];Wei He 2009[30];Yong Li 2012[26];Mary V.Relling 2004[41]
				Methylprednisolone/Prednisone	3	Masaaki Kuribayashi 2008[32];X.Y Yang 2007[36];Takeshi Asano 2003[44]
				Prednisone/Dexamethasone	1	Deborah French 2008[33]
			Primary disease			
				Hemoglobinopathies	1	Wei He 2009[30]
				Renal transplant	2	Masaaki Kuribayashi 2008[32];Takeshi Asano 2003[44]
				Acute lymphoblastic leukemia	2	Mary V.Relling 2004[41];Deborah French 2008[33]
				Systemic lupus erythematosus	1	X.Y Yang 2007[36]
ABCB1	Multinational	rs2032582	Allelic model		7	Yanqiong Zhang 2014[20];Yun Xue 2014[21];Wei He 2009[30];X.Y Yang 2007[36];Takeshi Asano 2003[44];Yong Li 2012[26];Mary V.Relling 2004[41]
			Heterozygous model		7	Yanqiong Zhang 2014[20];Yun Xue 2014[21];Wei He 2009[30];X.Y Yang 2007[36];Takeshi Asano 2003[44];Yong Li 2012[26];Mary V.Relling 2004[41]
			Homozygous model		7	Yanqiong Zhang 2014[20];Yun Xue 2014[21];Wei He 2009[30];X.Y Yang 2007[36];Takeshi Asano 2003[44];Yong Li 2012[26];Mary V.Relling 2004[41]
			Dominant model		7	Yanqiong Zhang 2014[20];Yun Xue 2014[21];Wei He 2009[30];X.Y Yang 2007[36];Takeshi Asano 2003[44];Yong Li 2012[26];Mary V.Relling 2004[41]
			Recessive model		7	Yanqiong Zhang 2014[20];Yun Xue 2014[21];Wei He 2009[30];X.Y Yang 2007[36];Takeshi Asano 2003[44];Yong Li 2012[26];Mary V.Relling 2004[41]
ABCB1	China	rs2032582	Allelic model		5	Yanqiong Zhang 2014[20];Yun Xue 2014[21];Wei He 2009[30];X.Y Yang 2007[36];Yong Li 2012[26]
			Heterozygous model		5	Yanqiong Zhang 2014[20];Yun Xue 2014[21];Wei He 2009[30];X.Y Yang 2007[36];Yong Li 2012[26]
			Homozygous model		5	Yanqiong Zhang 2014[20];Yun Xue 2014[21];Wei He 2009[30];X.Y Yang 2007[36];Yong Li 2012[26]
			Dominant model		5	Yanqiong Zhang 2014[20];Yun Xue 2014[21];Wei He 2009[30];X.Y Yang 2007[36];Yong Li 2012[26]
			Recessive model		5	Yanqiong Zhang 2014[20];Yun Xue 2014[21];Wei He 2009[30];X.Y Yang 2007[36];Yong Li 2012[26]
			Continuous variable			
				GA/GT vs GG	7	Yanqiong Zhang 2014[20];Yun Xue 2014[21];Wei He 2009[30];X.Y Yang 2007[36];Takeshi Asano 2003[44];Yong Li 2012[26];Mary

						V.Relling 2004[41]
				AA/TT/AT vs GG	7	Yanqiong Zhang 2014[20];Yun Xue 2014[21];Wei He 2009[30];X.Y Yang 2007[36];Takeshi Asano 2003[44];Yong Li 2012[26];Mary V.Relling 2004[41]
				Other vs GG	7	Yanqiong Zhang 2014[20];Yun Xue 2014[21];Wei He 2009[30];X.Y Yang 2007[36];Takeshi Asano 2003[44];Yong Li 2012[26];Mary V.Relling 2004[41]
			Type of steroids			
				Prednisolone	1	Yanqiong Zhang 2014[20]
				Prednisone	4	Yun Xue 2014[21];Wei He 2009[30];Yong Li 2012[26];Mary V.Relling 2004[41]
				Methylprednisolone/Prednisone	1	X.Y Yang 2007[36]
				Methylprednisolone/Prednisolone	1	Takeshi Asano 2003[44]
			Primary disease			
				Hemoglobinopathies	1	Wei He 2009[30]
				Renal transplant	1	Takeshi Asano 2003[44]
				Acute lymphoblastic leukemia	1	Mary V.Relling 2004[41]
				Systemic lupus erythematosus	1	X.Y Yang 2007[36]
ApoB	Multinational	rs693	Allelic model		4	Weibiao Fang 2011[27];Masaaki Kuribayashi 2008[32];Xiangdong Wei 2015[19];Ping Zeng 2014[23]
			Heterozygous model		4	Weibiao Fang 2011[27];Masaaki Kuribayashi 2008[32];Xiangdong Wei 2015[19];Ping Zeng 2014[23]
			Homozygous model		4	Weibiao Fang 2011[27];Masaaki Kuribayashi 2008[32];Xiangdong Wei 2015[19];Ping Zeng 2014[23]
			Dominant model		5	Weibiao Fang 2011[27];Masaaki Kuribayashi 2008[32];Xiangdong Wei 2015[19];Ping Zeng 2014[23];Tetsurou Hirata 2007[38]
			Recessive model		4	Weibiao Fang 2011[27];Masaaki Kuribayashi 2008[32];Xiangdong Wei 2015[19];Ping Zeng 2014[23]
ApoB	China	rs693	Allelic model		3	Weibiao Fang 2011[27];Xiangdong Wei 2015[19];Ping Zeng 2014[23]
			Heterozygous model		3	Weibiao Fang 2011[27];Xiangdong Wei 2015[19];Ping Zeng 2014[23]
			Homozygous model		3	Weibiao Fang 2011[27];Xiangdong Wei 2015[19];Ping Zeng 2014[23]
			Dominant model		3	Weibiao Fang 2011[27];Xiangdong Wei 2015[19];Ping Zeng 2014[23]
			Recessive model		3	Weibiao Fang 2011[27];Xiangdong Wei 2015[19];Ping Zeng 2014[23]
			Continuous variable			

				CT vs CC	4	Weibiao Fang 2011[27];Masaaki Kuribayashi 2008[32];Xiangdong Wei 2015[19];Ping Zeng 2014[23]
				TT vs CC	4	Weibiao Fang 2011[27];Masaaki Kuribayashi 2008[32];Xiangdong Wei 2015[19];Ping Zeng 2014[23]
				CT/TT vs CC	5	Weibiao Fang 2011[27];Masaaki Kuribayashi 2008[32];Xiangdong Wei 2015[19];Ping Zeng 2014[23];Tetsurou Hirata 2007[38]
			Type of steroids			
				Prednisone	3	Weibiao Fang 2011[27];Xiangdong Wei 2015[19];Ping Zeng 2014[23]
				Methylprednisolone/Prednisolone	2	Masaaki Kuribayashi 2008[32];Tetsurou Hirata 2007[38]
			Primary disease			
				Renal transplant	2	Masaaki Kuribayashi 2008[32];Tetsurou Hirata 2007[38]
ApoB	Multinational	rs1042031	Dominant model		4	Weibiao Fang 2011[27];Tetsurou Hirata 2007[38];Xiangdong Wei 2015[19];Ping Zeng 2014[23]
ApoB	China	rs1042031	Dominant model		3	Weibiao Fang 2011[27];Xiangdong Wei 2015[19];Ping Zeng 2014[23]
			Continuous variable			
				GA vs GG	4	Weibiao Fang 2011[27];Tetsurou Hirata 2007[38];Xiangdong Wei 2015[19];Ping Zeng 2014[23]
			Type of steroids			
				Prednisone	3	Weibiao Fang 2011[27];Xiangdong Wei 2015[19];Ping Zeng 2014[23]
				Methylprednisolone/Prednisolone	1	Tetsurou Hirata 2007[38]
			Primary disease			
				Renal transplant	1	Tetsurou Hirata 2007[38]
MTHFR	Multinational	rs1801133	Allelic model		3	Mary V.Relling 2004[41];Takeshi Asano 2004[42];A.Celik 2006[40]
			Heterozygous model		3	Mary V.Relling 2004[41];Takeshi Asano 2004[42];A.Celik 2006[40]
			Homozygous model		3	Mary V.Relling 2004[41];Takeshi Asano 2004[42];A.Celik 2006[40]
			Dominant model		4	Mary V.Relling 2004[41];Takeshi Asano 2004[42];A.Celik 2006[40];Deborah French 2008[33]
			Recessive model		3	Mary V.Relling 2004[41];Takeshi Asano 2004[42];A.Celik 2006[40]
			Continuous variable			
				TC vs CC	3	Mary V.Relling 2004[41];Takeshi Asano 2004[42];A.Celik 2006[40]
				TT vs CC	3	Mary V.Relling 2004[41];Takeshi Asano 2004[42];A.Celik 2006[40]
				TC/TT vs CC	4	Mary V.Relling 2004[41];Takeshi Asano 2004[42];A.Celik 2006[40];Deborah French 2008[33]

			Type of steroids			
				Prednisolone	1	A.Celik 2006[40]
				Prednisone	1	Mary V.Relling 2004[41]
				Methylprednisolone/Prednisolone	1	Takeshi Asano 2004[42]
			Primary disease			
				Acute lymphoblastic leukemia		
				Renal transplant	2	Takeshi Asano 2004[42];A.Celik 2006[40]
PAI-1	Multinational	rs1799768	Allelic model		3	Paolo Ferrari 2002[45];Yong Li 2012[26];Takeshi Asano 2004[42]
			Heterozygous model		3	Paolo Ferrari 2002[45];Yong Li 2012[26];Takeshi Asano 2004[42]
			Homozygous model		3	Paolo Ferrari 2002[45];Yong Li 2012[26];Takeshi Asano 2004[42]
			Dominant model		3	Paolo Ferrari 2002[45];Yong Li 2012[26];Takeshi Asano 2004[42]
			Recessive model		3	Paolo Ferrari 2002[45];Yong Li 2012[26];Takeshi Asano 2004[42]
			Continuous variable			
				4G5G vs 4G4G	3	Paolo Ferrari 2002[45];Yong Li 2012[26];Takeshi Asano 2004[42]
				5G5G vs 4G4G	3	Paolo Ferrari 2002[45];Yong Li 2012[26];Takeshi Asano 2004[42]
				4G5G/5G5G vs 4G4G	3	Paolo Ferrari 2002[45];Yong Li 2012[26];Takeshi Asano 2004[42]
			Type of steroids			
				Prednisone	2	Paolo Ferrari 2002[45];Yong Li 2012[26]
				Methylprednisolone/Prednisolone	1	Takeshi Asano 2004[42]
			Primary disease			
				Renal transplant	2	Paolo Ferrari 2002[45];Takeshi Asano 2004[42]
